# Clinical Application of MicroRNA Testing in Neuroendocrine Tumors of the Gastrointestinal Tract

**DOI:** 10.3390/molecules19022458

**Published:** 2014-02-21

**Authors:** Caterina Vicentini, Matteo Fassan, Edoardo D’Angelo, Vincenzo Corbo, Nicola Silvestris, Gerard J. Nuovo, Aldo Scarpa

**Affiliations:** 1 ARC-Net Research Centre, University and Hospital Trust of Verona, Verona 37134, Italy; 2 Department of Pathology and Diagnostics, University and Hospital Trust of Verona, Verona 37134, Italy; 3 Medical Oncology Unit, National Cancer Institute “Giovanni Paolo II”, Bari 70124, Italy; 4 Comprehensive Cancer Centre, Ohio State University, Columbus, OH 43210, USA

**Keywords:** miRNAs, neuroendocrine tumors, pancreas, gastrointestinal tract, *in situ* hybridization

## Abstract

It is well documented that dysregulation of microRNAs is a hallmark of human cancers. Thus, this family of small non-coding regulatory molecules represents an excellent source of sensitive biomarkers. Unique microRNAs expression profiles have been associated with different types and subsets of gastrointestinal tumors including gastroenteropancreatic neuroendocrine tumors (GEP-NETs). GEP-NETs are a heterogeneous group of epithelial neoplasms with neuroendocrine differentiation. At present, early detection and surgical resection of GEP-NETs represent the best chance for a cure. Thus, clinically useful biomarkers for GEP-NETs that strongly correlate with early detection are urgently needed. The purpose of this review is to summarize the role of miRNAs in GEP-NET carcinogenesis and their possible use as novel diagnostic, prognostic and predictive biomarkers.

## 1. Introduction

Gastroenteropancreatic neuroendocrine tumors (GEP-NETs) represent a heterogeneous group of epithelial neoplasms with neuroendocrine differentiation that can arise in all the organs of the gastrointestinal system and pancreas. Significant clinico-pathological differences exist among the different subtypes of GEP-NETs. The most common histologic subtype, accounting for 85% of GEP-NETs, is the well differentiated form [1,2,3,4]. However, its clinical behavior varies from indolent and slow growing to aggressive, metastatic, and rapidly fatal; at present there is no histologic feature which can separate these very disparate clinical outcomes. The other main category of GEP-NETs is the poorly differentiated neuroendocrine carcinoma that carries a poor prognosis [1,2,3,4].

GEP-NETs are defined as functioning (syndromic) or nonfunctioning (nonsyndromic), depending on the presence of a syndrome related to inappropriate hormone secretion [1]. Nonsyndromic GEP-NET patients usually present with mass-related symptoms or metastatic disease in the absence of a noteworthy past medical history [2,3]. As a result, tumor diagnosis can be delayed for years, and a substantional number of patients with nondyndromic GEP-NETs are admitted for medical workout with unresectable disease [1,2,3].

At present, the early detection and the subsequent surgical resection represents the best chance for a definitive cure [4]. The most widely used biomarker to determine disease burden and monitor response to treatment is serum chromogranin A. However, chromogranin A has a relatively high false negative rate and shows poor correlation with disease severity [4].

The rarity of the disease and the fact that many patients with GEP-NETs show an indolent course has discouraged significant research efforts for this disease [5]. However, due to the advent of the targeted therapy era, the treatment options and biologic understanding of GEP-NETs has increased notably in recent years. For example, recent molecular studies investigated the genomic landscape of these tumors [6,7,8,9,10,11]. This has resulted in the discovery of mutations and expression anomalies in genes and pathways such as the *PI3K*/Akt/mTOR, *DAXX*/*ATRX*, and *MEN1*, which in turn may lead to new and better prognostic biomarkers as well as future candidates for targeted therapies [6,7,8,9,10,11]. Indeed, the VEGF pathway inhibitor sunitinib, and the mTOR inhibitor everolimus have been successfully applied in the treatment of advanced pancreatic NETs [12,13].

Among these potential new diagnostic and therapeutic targets, microRNAs represent a recently uncovered class of small and endogenous non-coding RNAs [14]. MiRNA dysregulation is a hallmark of human cancer and miRNA genome-wide studies in different tumors have highlighted that selective groups of distinct miRNAs are commonly dysregulated in specific types of human malignancies. The dysregulated miRNAs, in turn, are often correlated with diagnosis, staging, progression, prognosis and response to clinical therapies [15]. This review briefly summarizes the role of miRNAs in GEP-NET carcinogenesis, and highlights their potential use as novel diagnostic, prognostic and predictive biomarkers.

## 2. Biogenesis and Function of miRNAs

MiRNAs are a class of endogenous, small (19 to 25 nucleotides), non-coding RNAs that modulates the expression of at least one third of protein-coding genes. As a result, miRNAs regulate all aspects of cell proliferation, differentiation and function, and their discovery has shed new light on the mechanisms of both cell physiology and human pathology [16].

MiRNA genes are first transcribed by RNA polymerase II in the nucleus into long transcripts called primary miRNAs (pri-miRNAs) [17]. These are characterized by a 5-cap structure (7MGpppG) as well as a 3-end poly(A) tail. MiRNA genes may be transcribed as monocistronic or polycistronic (e.g., *miR-17-92-1* cluster) messenger RNAs and their transcriptional regulation is controlled by the host tissue genes, which make miRNA expression tissue specific [18]. Clustered miRNAs might be transcribed from a single transcription unit as polycistronic primary-miRNA.

The pri-miRNAs are processed by the nuclear RNase III Drosha and its cofactor DGCR8 (DiGeorge syndrome critical region gene 8). This complex generates 60-100 nucleotides precursor miRNA (pre-miRNAs) products, which locally fold into stable secondary stem-loop structures [19,20]. Pre-miRNAs are exported to the cytoplasm by Ran-GTP-dependent transporter Exportin 5. In the cytoplasm, the pre-miRNAs are processed by a second RNase III enzyme, Dicer, which cuts off the terminal loop and releases transitory double-stranded RNA duplexes called miRNA/miRNA* [21].

The two potentially functional strands released by Dicer are separated by helicases, and the mature strand is incorporated into the RNA-induced silencing complex (RISC) [17]. The guide strand of the duplex, which corresponds to the mature miRNA, is then incorporate into the RISC, while the other strand is usually degraded. The so called miRNA* was initially thought to be the strand subjected to degradation; however, more recent evidence suggests that it does not simply represent a non-functional bioproduct of miRNA biogenesis, but it can be selected as a functional strand and play significant biological roles.

In the RISC, miRNAs negatively regulate gene expression at the post transcriptional level by base pairing to partially complementary sites on target messenger RNAs (mRNAs), usually in the 3' untranslated region (UTR). Binding of a miRNA to the target mRNA typically leads to translational repression and exonucleolytic mRNA decay, although highly complementary targets can be cleaved endonucleolytically [22]. Additional findings suggest, however, that both miRNA biogenesis and function are more complex than previously expected [17]. MiRNAs can increase the translation of a target mRNA by recruiting protein complexes to the AU-rich elements of the mRNA, or they can indirectly increase the target protein output by de-repressing mRNA translation by interacting with proteins that block the translation of the target gene [23,24]. Recent evidences indicate that miRNAs can cause global protein synthesis by enhancing ribosome biogenesis, or switch the regulation from repression to activation of target gene translation in conditions of cell cycle arrest [23,25].

Another important mechanism of miRNAs function is their influence on neighboring cells and at more distant sites within the body in a hormone-like fashion [17]. In fact, miRNAs, together with RNA-binding proteins (such as Nucleophosmin 1 and Argonaute 2), can be packaged and transported extracellularly by exosomes or microvesicles [26,27,28,29]. Circulating miRNAs enter the bloodstream and are taken up by the recipient cells via endocytosis and further bind to intracellular proteins such as Toll-like receptors (TLRs) [26]. The circulating miRNAs can, in turn, be detected in plasma or other body fluids in much the same way other classic markers of cell dysfunction such as troponin or CEA can be used as markers of cardiac infarcts/colon oncogenesis.

## 3. MiRNAs and Human Cancer

The first evidence of miRNAs involvement in human cancer occured in 2002 through the characterization of chromosome 13q14 in chronic lymphocytic leukemia (CLL) [30]. This chromosomal region, frequently deleted in CLL, does not contain any protein-coding tumor suppressor gene, but two microRNA genes, *miR-15a* and *miR-16-1*, expressed in the same polycistronic RNA [30]. The fact that miRNA-15 and 16 can regulate bcl-2 expression provided the genetic basis for the marked bcl-2 overexpression and resultant loss of apoptosis typical of CLL. After this seminal discovery, a myriad of reports have irrefutably demonstrated that aberrant miRNA expression is a hallmark of human disease, including cancer. The cause of the widespread differential expression of miRNA genes between tumor and normal cells can be explained by different mechanisms including chromosomal alterations, DNA point mutations, epigenetic mechanisms or alterations in the machinery responsible for miRNA production [31,32,33,34,35,36]. Like the coding genes, miRNAs can be either overexpressed or underexpressed and they can act as tumor-suppressors or oncogenes based on the downstream target that the miRNA controls [15].

**Figure 1:**
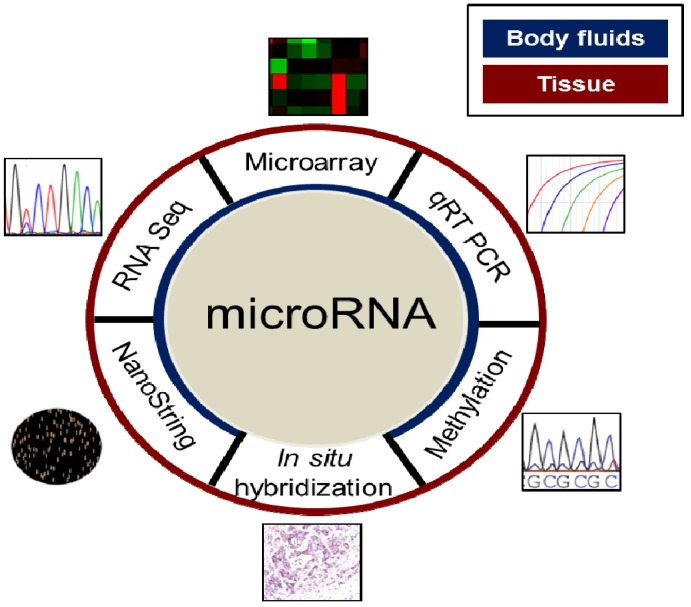
Overview of the different molecular techniques applied in the study of miRNA dysregulation in human pathology. All techniques could be applied to tissue sample (**red** **circle**; both fresh and formalin-fixed paraffin-embedded) and body fluids samples (**blue circle**; mainly urine and plasma).

As noted, miR-15a and -16-1 were the first described tumor-suppressor miRNAs. Their loss, by releasing the inhibition upon tumor-promoting genes, such as *BCL2*, *BMI1*, *CCND2* and *CCND1*, promotes cell growth and tumor progression [16]. In comparison, *miR-21* is highly up regulated in the majority of human cancer tissues and by repressing pro-apoptotic genes, such as *PTEN* or *PDCD4*, stimulates proliferation and tumor initiation (Figure 1) [16]. It is important to note that some miRNAs can behave like oncogenes in one cell type and as tumour suppressors in other cells. For example, the over expression of *miR-221* in hepatocellular carcinoma exerts an oncogenic function by downregulating the expression of *PTEN*, but in erythroblastic leukaemia it acts as a tumour suppressor by reducing the expression of the *KIT* oncogene [15,17].

The recent development of different high-throughput miRNA profiling technologies has allowed the characterization of the miRNA expression profile for many human malignancies. MiRNA expression profiles differ depending upon cell, tissue, and disease, and miRNA profiling can accurately classify the different tumors according to the tissues of origin, which can be especially helpful in the case of poorly differentiated carcinomas of unknown origin [37,38]. MiRNA profiling can also discriminate different subtypes of a particular cancer or even specific oncogenic abnormalities [37,38,39,40]. From the clinical point of view, large profiling studies have proven the utility of miRNA fingerprints in the definition of tumor prognosis and treatment of cancer when combined with standard gene profiling [16].

## 4. Evaluation of MiRNAs in the Clinical Setting

The use of the traditional histological examination in the diagnosis and classiﬁcation of cancers can be limited by the availability of adequately preserved tissue and the possibility of subjective interpretation by pathologists [31]. Unfortunately, molecular profiling focusing on DNA or mRNA from archived formalin-fixed paraffin-embedded (FFPE) samples is patchy, which makes it difficult to consistently integrate molecular data with histological information. In contrast to mRNAs, miRNAs are long-living *in* *vivo* and very stable *in* *vitro*, which enables miRNA profiling techniques to be extremely sensitive, objective and standardized tools even in formalin fixed, paraffin embedded tissues. Indeed, miRNAs can be extracted from various specimen types including fresh or FFPE tissues, and body fluids such as plasma, serum, urine, and sputum. Although there is currently no gold standard for measuring miRNA expression, several different methods can be successfully applied (Figure 1) [41].

Many reports have already demonstrated the excellent reproducibility and accuracy of miRNA expression profiling in archived specimens. Moreover, in FFPE specimens, miRNA expression can also be visualized at cellular/subcellular level by *in situ* hybridization, and this particular feature makes miRNAs potentially suitable for supporting routine diagnostic histopathological practice [42]. Another important potential of miRNAs as biomarkers is their detection in body fluids (Figure 1), which relies mainly on their high stability and resistance to storage and handling [15]. Specific serum and plasma miRNA expression patterns have been identified in tumor patients and they can provide fingerprints for various diseases [15,16]. Correlations between circulating miRNA levels and the response to a given anticancer agent have also been observed and may be useful in predicting patterns of resistance and sensitivity to particular drugs [15,16]. The stability, low complexity, and the availability of inexpensive methods of detection and profiling could make extracellular miRNAs ideal biomarkers.

## 5. MiRNAs and Novel Therapeutic Strategies

Each step of miRNA generation and function, both intracellular and endocrine, can potentially be therapeutically targeted. Preclinical models have consistently highlighted the efficacy of miRNA based therapies, either alone or in combination with current targeted therapies [14]. The main advantage of miRNA-based strategies is that miRNAs target several coding or non-coding genes that can be involved in a specific pathway or in redundant pathways involved in cancer development. In other words, the ability of miRNAs to target genes that are implicated in the same pathway and/or in interacting pathways provides the biological rationale for the use of a small number of miRNAs to achieve a broad silencing of pro-tumor pathways.

The therapeutic application of miRNAs involves two strategies: miRNA reduction and miRNA replacement. miRNA reduction is directed against a gain of function and seeks to inhibit oncogenic miRNA by: (i) small-molecule inhibitors acting on regulation of miRNAs expression at the transcriptional level; (ii) antisense oligonucleotides that bind to complementary miRNAs and induce either duplex formation or miRNA degradation; (iii) miRNA masking using molecules complementary to the 3'-UTR of target miRNA, resulting in competitive inhibition of downstream target effects, and (iv) miRNAs sponges, oligonucleotide construct with multiple complementary miRNA binding sites to target miRNAs [43]. The second strategy, miRNA replacement, is directed against a loss of function and involves the reintroduction of a tumor suppressor miRNA to restore a loss of function by introducing systemic miRNAs (miRNA mimics) or inserting genes coding for miRNAs into viral constructs [43,44,45]. Even though these two approaches are promising, there are two main problems that have hindered the development and the efficacy of miRNA-based treatments so far. The first one is represented by the necessity to obtain a tissue-specific delivery and develop a more efficient cellular uptake of synthetic oligonucleotides to get constant target inhibition. The second important problem regards the relative low efficiency of miRNAs in body fluids or tissues that seek to recover lost function or block a tumor enhancing miRNA, which has been partially overcome by the use of locked nucleic acid (LNA) constructs [14]. The most successful application of the so-called “LNA anti-miR” has culminated in the first miRNA-based clinical trial for the treatment of hepatitis C virus infection by targeting miR-122 with a LNA-antimiR (miravirsen or SPC3649; Santaris Pharma, Hørsholm, Denmark) [46].

## 6. MiRNAs Dysregulation in GEP-NETs

In contrast to many other tumor types, very little is known about microRNA expression patterns in GEP-NETs. However, a significant number of oncogenic and suppressor miRNAs have been identified in pancreatic (PanNETs) and ileal GEP-NETs, which further support the possible use of specific miRNAs signatures to predict clinical outcome in neuroendocrine tumors [47,48,49,50]. In our report, we investigated global microRNA expression signatures of normal pancreas, Langerhans’ islets, PanNETs, and pancreatic acinar carcinomas [47]. A three microRNA signature, corresponding to the overexpression of the two highly homologous miR-103 and miR-107 together with the lack of expression of miR-155, was able to discriminate significantly between tumors *versus* non-tumor tissues in our study. Moreover, a set of 10 miRNAs (miR-125a, miR-99a, miR-99b, miR-125b-1, miR-342, miR-130a, miR-132, miR-129-2, miR-125b-2) allowed the discrimination of PanNETs from acinar tumors. This specific miRNA signature is possibly associated with either normal endocrine differentiation or endocrine tumor genesis.

**Figure 2:**
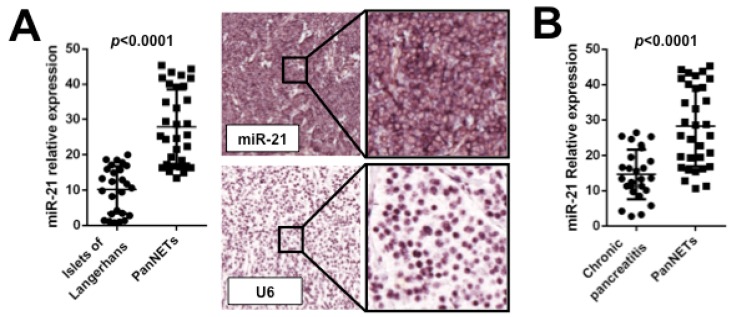
miR-21 is significantly overexpressed in pancreatic neuroendocrine tumors and it is detectable in patients’ plasma samples. (**A**) Pancreatic neuroendocrine tumors (PanNETs) usually show a significantly higher miR-21 expression in comparison to normal pancreatic Langerhans islets (qRT-PCR). This overexpression is confirmed by *in* *situ* hybridization (upper panel: a PanNET showing strong miR-21 staining; lower panel: same case stained with the positive control U6). (**B**) In PanNET patients, circulating cell-free plasma miR-21 is overexpressed in comparison to chronic pancreatitis patients and could be used as a novel diagnostic biomarker.

Among PanNETs, miR-204 was primarily expressed in insulinomas (well-differentiated), and correlated with immunohistochemical expression of insulin. On the other hand, miR-21 overexpression was associated with high Ki67 proliferation index and the presence of liver metastases [47] (Figure 2). The fact that *PTEN* is one of miR-21 targets further support the increasing evidence of mTOR pathway involvement in NETs pathogenesis.

Matthaei and colleagues from Johns Hopkins Hospital identified a miRNAs expression pattern including nine miRNAs (miR-24, miR-30a-3p, miR-18a, miR-92a, miR-342-3p, miR-99b, miR-106b, miR-142-3p, and miR-532-3p) derived from the analysis of selected miRNAs in cyst fluid samples, which successfully discriminated cystic forms of PanNETs from other pancreatic cystic lesions, such as intraductal papillary mucinous neoplasms (IPMN) [51]. Another group from the Johns Hopkins investigated cell-free circulating miRNA profiles on a relatively large series of patients with pancreatic adenocarcinoma, healthy controls, subjects with chronic pancreatitis, and PanNETs [52]. The serum down-regulation of miR-1290 had the best diagnostic performance in discriminating PanNETs from adenocarcinomas (AUC area of 0.80; 0.67–0.93). Other significantly down-regulated serum miRNAs in PanNETs patients were miR-584, miR-1285, miR-550-002410, and miR-1825 [52].

Two relatively recent miRNA expression profile studies provided evidence of miRNA dysregulation in ileal NET progression [48,49]. Rubel and colleagues from the Mayo Clinic associated miRNA-133a downregulation to progression from primary to metastatic carcinoid neoplasms, suggesting that it may have an important role in ileal NETs development and progression with use for diagnosis and/or prognosis [48]. The same group in collaboration with Uppsala University investigated miRNA expression in well-differentiated small intestinal NETs. Nine miRNAs were significantly dysregulated: five (miR-96, miR-182, miR-183, miR-196, and miR-200) were upregulated during tumour progression, whereas four (miR-31, miR-129-5p, miR-133a, and miR-215) were significantly downregulated in ileal NETs [49]. Finally, a single case of colonic NET has been profiled by Illumina high-throughput sequencing, and demonstrated a total of 38 miRNAs significantly dysregulated compared to the normal colonic mucosa samples [53].

## 7. Future Directions and Conclusions

The use of miRNAs as diagnostic and prognostic biomarker for cancer treatment is rapidly increasing. Moreover, many researchers are exploring the potential use of these small non-coding RNA as therapeutic molecules. Only limited data on miRNAs’ role in GEP-NETs has been accumulated so far. The challenges ahead lie in the reliable identification of progression-specific targets to enable molecular testing in the clinical management of GEP-NETs as well as the development of safe and specific methods of delivery of miRNAs for innovative GEP-NETs treatments.
